# Effect of Torso and Breast Characteristics on the Perceived Fit of Body Armour Systems Among Female Soldiers: Implications for Body Armour Sizing and Design

**DOI:** 10.3389/fspor.2022.821210

**Published:** 2022-03-09

**Authors:** Celeste E. Coltman, Brooke R. Brisbine, Richard H. Molloy, Julie R. Steele

**Affiliations:** ^1^Faculty of Health, University of Canberra Research Institute for Sport and Exercise, University of Canberra, Canberra, ACT, Australia; ^2^Human Systems Integration, Land Division, Defence Science and Technology Group, Department of Defence, Melbourne, VIC, Australia; ^3^Biomechanics Research Laboratory, University of Wollongong, NSW, Australia

**Keywords:** anthropometry, equipment design, protective equipment, female soldier, body armour

## Abstract

This study aimed to provide normative data characterising the torsos and breasts of female soldiers and to determine which torso and breast anthropometric measurements contributed to reports of poor body armour fit. Ninety-seven female Australian Army soldiers completed a questionnaire about their experience with current-issue body armour, including perceptions of fit. Participants also attended a single testing session where we took a three-dimensional scan of their breasts and torso and collected several anthropometric measurements to characterise their torso size and shape. Sixteen of the 22 breast and torso measurements collected were significantly related to the perceived fit of current-issue body armour systems. To improve perceptions of fit for female soldiers and, in turn, reduce movement interference, discomfort, and barriers to occupational performance, future body armour systems should cater to the wide range of female breast and torso shapes and sizes.

## Introduction

Military body armour functions to protect the vital thoracoabdominal organs of soldiers from ballistic, fragmentation and stab threats (Choi et al., 2016; Laing and Jaffrey, 2019). Body armour, however, can introduce integration issues between the human user and the system, as well as present a mass burden (Knapik et al., 2004). These integration issues are amplified if the body armour system is ill-fitting. Soldiers wear body armour during operational deployment and training or field-based exercises for 5.2–6.4 ± 4.7 h per day (Coltman et al., 2020). It is therefore vital that a body armour system (comprising front and rear hard and soft ballistic plates encased in a carrier) interfaces appropriately with a soldier's torso (i.e., fits correctly) and integrates well with other elements of a soldier's combat ensemble (Furnell et al., 2017). Proper fit will maximise protection and maintain coverage requirements over this long duration of wear (Laing and Jaffrey, 2019). The fit of body armour also has important implications for job and combat performance. That is, poor-fitting body armour can impede a soldier's ability to run efficiently, to shoulder and fire a rifle, to manoeuvre in and out of a vehicle and to complete other mission essential tasks (Mitchell et al., 2010; Choi et al., 2016, 2018; Coltman et al., 2020, 2021a). Consequently, designing and sizing body armour to properly fit all soldiers is vitally important for military organisations to achieve correctly.

Previous research has revealed that most female soldiers are dissatisfied with the fit of their current body armour systems (Epstein et al., 2013; Toma et al., 2016; Coltman et al., 2020, 2021a; Davis et al., 2020). Specifically, questionnaire data related to the fit of body armour was completed by 147 female Australian Defence Force (ADF) soldiers in both combat and non-combat roles. Of the women surveyed, 68, 56, and 12%, respectively, found the body armour to be ill-fitting, too large, and too small (Coltman et al., 2020). Similarly, researchers investigating the fit and function of body armour in the United Kingdom found that female soldiers reported numerous instances of discomfort, particularly at the hip (Davis et al., 2020). In the same study 29–59% of participants experienced task interference during a range of basic job roles (Davis et al., 2020). Another group found that integration between a soldier's bra and body armour was a source of substantial discomfort (Malbon et al., 2020). The notion of sex-specific discomfort with body armour was supported by findings from an Australian study, which revealed that limited adjustability of the system, insufficient space for breasts and oversized length and width were all common problems reported by female soldiers (Coltman et al., 2021a). Female soldiers now comprise 10.4–17.5% of Defence Force populations in Australia, Canada, New Zealand, United Kingdom and United States of America (Defence People Group., 2017; Ministry of Defence., 2018; Mark, 2019; Service Women's Action Network., 2019; Government of Canada., 2020). Ensuring that body armour is appropriately sized and designed to enable female users to undertake their occupational roles is essential to fostering women's participation and inclusion in Defence.

A primary source of poor body armour fit for female soldiers is how it is sized and designed (Epstein et al., 2013; Toma et al., 2016; Coltman et al., 2020, 2021a; Malbon et al., 2020). Although several anthropometric surveys have been conducted on global soldier populations to inform the design and sizing of protective equipment, military body armour is commonly issued to soldiers in a limited, unisex sizing range (Todd, 2007). Moreover, the dimensions and specifications of body armour systems are traditionally based upon male anthropometric data (Todd, 2007; Epstein et al., 2013; Toma et al., 2016). This is likely a function of body armour primarily being designed for combat soldiers. Until 2011, women in the ADF were ineligible to enlist in combat coded roles (Wadham et al., 2018) and therefore only men served in combat. Furthermore, women comprise only a small percentage of the soldier population in anthropometric surveys and there is a lack of breast specific measurements included in such surveys to better inform body armour designs (Edwards et al., 2014). Given that women have different torso shapes and more developed breast tissue compared to men (Edwards et al., 2014; Gordon et al., 2014), designs that have not specifically accounted for female anthropometry are unlikely to properly fit female soldiers. Poor personal protective equipment (PPE) fit has also been observed among female workers in a range of historically male-dominated occupations such as firefighting and construction (Barker et al., 2012; Onyebeke et al., 2016). Evidence also highlights the sex disparity in PPE design affecting occupational task performance (Park and Hahn, 2014). No previous research, however, has specifically investigated which breast and torso characteristics contribute to poor fit of body armour systems for female soldiers. Such information is required to understand the association between anthropometric torso dimensions and equipment fit in body armour design and sizing. This exploratory study, therefore, aimed to: (i) provide normative data characterising the torso and breast size and shape of female soldiers to inform evidence-based design modifications to current-issue body armour, and (ii) determine which breast characteristics and torso anthropometric measurements contributed most to poor body armour fit reported by female soldiers. It was hypothesised that female soldiers would display a wide range of breast and torso characteristics and that these characteristics would differ between soldiers who reported that their body armour was too small, too large or a good fit.

## Materials and Methods

### Participants

**Figure 1:**
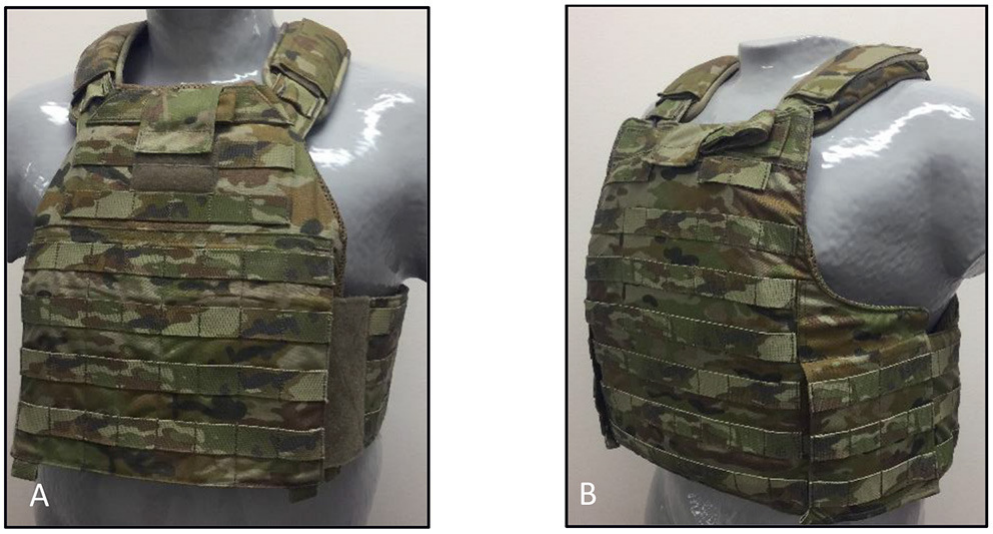
Current Australian body armour is the Tiered Body Armour System (TBAS) V4.4, issued to personnel in either Tier 2 **(A)** or Tier 3 **(B)**. The same size hard plate and soft armour inserts are used in both systems. Tier 3 affords slightly greater protection to the solider, as shown by increased soft armour coverage on the side wings.

Ninety-seven female soldiers (Table 1) from various units within the Australian Army volunteered to participate in the present study. Potential participants were excluded if they had epilepsy that might be induced by the flashing light of the three-dimensional scanner (described below). All participants had experience wearing ADF issue body armour [Tiered Body Armour System (TBAS) V4.4 Tier 2 or Tier 3; Figure 1] and provided written informed consent before being assigned to attend a single test session, scheduled either in May or June 2019. Ethical approval for the study was obtained from the Defence Science and Technology Group Low Risk Ethics Panel (LD 07-351) and cross-institutional approval was obtained from the University of Canberra Human Research Ethics Committee (Project ID: 2013).

**Table 1:** Participant characteristics (*n* = 97).

**Characteristic**	**Participant** **data**
Age (mean ± SD)	25.6 ± 7.4 years
BMI (mean ± SD)	25.4 ± 3.3 kg/m^2^
Underweight (<18.5 kg/m^2^)	0% (*n* = 0)
Healthy weight (18.5–24.9 kg/m^2^)	54% (*n* = 52)
Overweight (25–29.9 kg/m^2^)	38% (*n* = 37)
Obese (>30 kg/m^2^)	8% (*n* = 8)
Bra band size (mode; range)	10 (8–16)
Bra cup size (mode; range)	C (A–H)
Combat-arms employment categories	23% (*n* = 22)
Non-combat arms employment categories	77% (*n* = 75)
Years in Army (mean ± SD)	4.3 ± 4.8 years
TBAS V4.4 Tier 2 users	36% (*n* = 35)
TBAS V4.4 Tier 3 users	64% (*n* = 62)
Body armour wear duration during training (mean ± SD)	6.4 ± 4.7 h/day
Body armour wear duration during operations (mean ± SD)	5.2 ± 4.7 h/day

### Torso Characteristics

To characterise each participant's torso dimensions, a series of anthropometric measurements (described in Table 2) were collected according to procedures outlined by the International Standards for Anthropometric Assessment (Marfell-Jones et al., 2012). Measurements were chosen based on their relevance to the design of military body armour. This included anthropometric dimensions specifically recommended by the Australian Warfighter Anthropometry Survey (AWAS) as being pertinent to body armour (Edwards et al., 2014) and several additional measurements from AWAS deemed potentially relevant by subject matter experts within the Australian Army. Data were collected while participants wore long pants and a bra only. Each measurement was repeated three times, with the mean of the three measurements recorded in centimetres (cm).

**Table 2:** The anthropometric measurements collected during the current study, including a brief description of the protocol associated with each measurement.

**Measurement**	**Description of how the measurement was taken**
Stature (cm)^*^	The participant stood against a portable stadiometer (model: 213, Seca Corp., Maryland, USA) with her feet together and heels against the back of the stadiometer. With her head in the Frankfort plane, the participant was instructed to take a deep breath in while the researcher applied a gentle lift through the mastoid processes and then placed the headboard of the stadiometer firmly down on the participant's vertex.
Body Mass (kg)^*^	Body mass (recorded to the nearest 0.1 kg) was measured while each participant stood on calibrated body mass scales (model: RD 545, Tanita, Illinois, USA) without wearing shoes and socks.
Iliocristale Height (cm)^*^	The most lateral edge of the iliac crest on the participant's left ilium was palpated and marked. The participant was then instructed to stand up straight while the vertical distance between the floor (standing surface) and her iliocristale marking was measured using an anthropometer (Siber-Hegner, Zurich, Switzerland).
Waist Height (cm)^*^	The participant's waist was palpated and marked at the level of the narrowest point between her lower costal (10th rib) border and the iliac crest on the left side of her body. The participant was then instructed to stand up straight while the vertical distance between the floor (standing surface) and her waist marking was measured using an anthropometer.
Suprasternale Height (cm)^*^	The participant's suprasternal notch was palpated and marked. The participant was instructed to stand up straight and the vertical distance between the floor (standing surface) and her suprasternal marking was measured using an anthropometer.
Front Length (cm)^*^	The suprasternale height measurement was subtracted from the iliocristale height measurement to calculate front length.
Chest Depth (cm)^*^	With the participant in a standing position, a sliding caliper (Campbell 20, Rosscraft International, British Columbia, Canada) was placed at the level of her mesosternale (anteriorly) and on the spinous process of her vertebra (posteriorly) at the horizontal level of the mesosternale. The participant was instructed to breathe normally and the measurement was taken at the end of tidal expiration.
Chest Breadth (cm)^*^	The participant assumed a relaxed standing position with her arms abducted. The sliding caliper was positioned at the level of the mesosternale (anteriorly) and the distance was recorded between the most lateral aspect of the thorax at the end of tidal expiration.
Bi-acromial Breadth (cm)^*^	The participant assumed a standing position with her arms hanging by her sides while the distance between the most lateral points of her acromion processes was measured.
**Neck Circumference (cm)**	The participant assumed a relaxed standing position with her arms hanging by her sides and a tape measure (W606PM, Lufkin, United States) was applied around the base of her neck.
Waist Circumference (cm)^*^	The participant assumed a standing position with her arms folded across her thorax. The circumference was measured at the level of the narrowest point between the 10th rib and iliac crest at the end of normal expiration.
**Hip Circumference (cm)**	The participant assumed a relaxed standing position with her arms folded across her thorax. The circumference was taken at the greatest posterior protuberance of her buttocks.
Over-Bust Chest Circumference (OBCC) (cm)^*^	The participant assumed a standing position with her arms hanging by her sides and slightly abducted. The girth was taken at the level of the widest point of her bust at the end of a normal expiration.
Under-Bust Chest Circumference (UBCC) (cm)^*^	The participant assumed a standing position with her arms hanging by her sides and slightly abducted. The girth was taken at the level directly below her bust at the end of a normal expiration.

* *indicates measurements that were deemed relevant to the design of body armour in the Australian Warfighter Anthropometry Survey (Edwards et al., 2014)*.

### Breast Characteristics

A three-dimensional scanning protocol was used to characterise each participant's breasts. Adhesive markers (~1 cm in diameter) were placed on the participant's bra and breast to outline the boundaries of each breast (Table 3A). The participant was instructed to stand up straight and look forward with the heel of her hands resting on her hips. The breasts and torso of each participant were then scanned using a hand-held three-dimensional scanner (Artec^TM^ Eva 3D Scanner, Artec Group, San Jose, USA) while she was wearing a standardised encapsulation-type bra (New Legend Underwire sports bra, Berlei, Wentworthville, NSW, Australia). The test bra was professionally fitted (McGhee and Steele, 2010) and chosen for its ability to separate the breasts and enable clear visualisation of a defined breast border. From this scan, a three-dimensional model of the breast was created (Tables 3E,F). Although three-dimensional breast characteristics are usually quantified while women are bare-breasted (Coltman et al., 2018, 2021b; McGhee and Steele, 2019), ethical limitations necessitated a modified protocol in the present study. As female soldiers wear a bra when using body armour systems, it was deemed appropriate to calculate breast characteristics while participants wore a standardised bra. The standardised bra was chosen for its ability to separate a participant's breasts without drastic compression so that all anatomical landmarks required to calculate the breast measurements were clearly visible. Importantly, the standardised bra was worn only during scanning and therefore did not have any effect on the participants' ratings of perceived body armour fit, which was answered in relation to whichever bra style and size they normally wore. This scanning protocol is also consistent with recommendations for breast measurements taken to inform the design of protective equipment worn external to a bra (Brisbine et al., 2020a) and previous research undertaken by the research team (Coltman et al., 2021b).

**Table 3:** A description of how each breast measurement was digitally calculated, as well as a visual depiction of the measurement in Geomagic.

**Measurement**	**Description of how the measurement was calculated in Geomagic**	**Visual depiction of the measurement**
**Breast Volume (mL)**	From each participant's scan, a three-dimensional, isolated model of each breast was created by outlining the breast **(A)**, removing the breast from the torso **(B)** and attaching it to the corresponding anterior chest wall (whose curvature approximated the superficial surface of the pectoralis major muscle; **C,D**). These steps were performed to create a closed three-dimensional breast model **(E,F)**, from which breast volume (mL) was calculated.	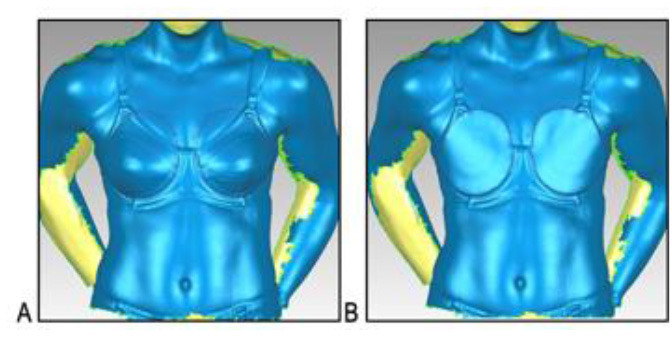
**Breast Surface Area (cm** ^**2**^ **)**	Using the closed three-dimensional breast model **(E,F)**, the surface area (cm^2^) of each participant's right and left breast was calculated.	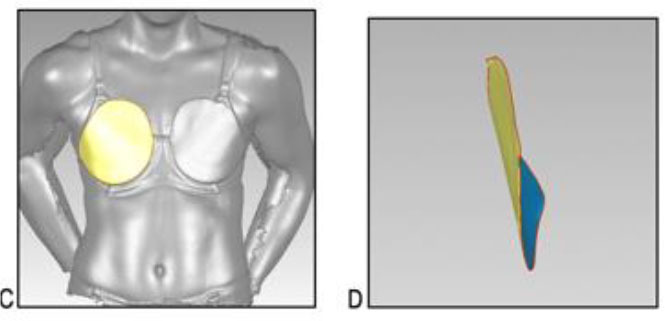
**Anterior Breast Projection (mm)**	Using the closed three-dimensional breast model **(E,F)**, the distance from the posterior breast wall to the most anterior point of the breast was calculated.	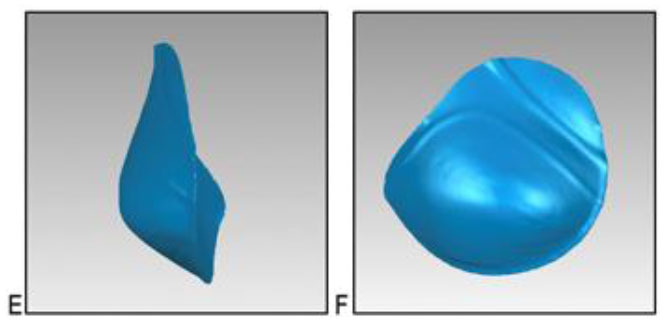
**Breast Length (mm)**	The linear distance (mm) between the inferior and superior borders of each participant's right and left breast was measured at the longest vertical point.	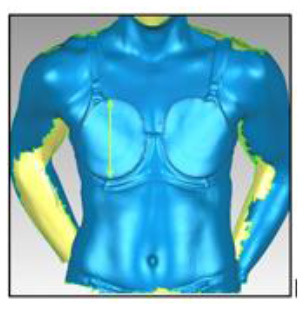
**Breast Width (mm)**	The linear distance (mm) between the medial and lateral borders of each participant's right and left breast was measured at the widest point.	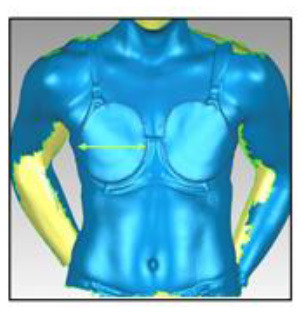
**Sternal Notch to Nipple Distance (mm)**	The linear distance (mm) from the sternal notch (where a marker had been placed before scanning) to the nipple of each participant's right and left breast was measured.	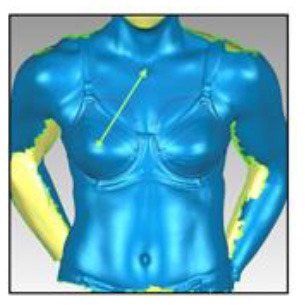
**Sternal Notch to Superior Breast Distance (mm)**	The perpendicular distance (mm) between the sternal notch and a horizontal line drawn across the torso at the level of the superior border of each participant's right and left breast was measured.	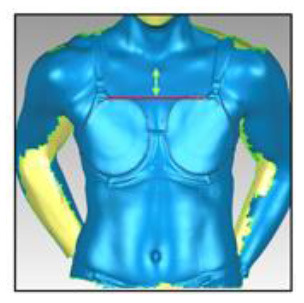
**Sternal Notch to Inferior Breast Distance (mm)**	The perpendicular distance (mm) between the sternal notch and a horizontal line drawn across the torso at the level of the superior border of each participant's right and left breast was measured.	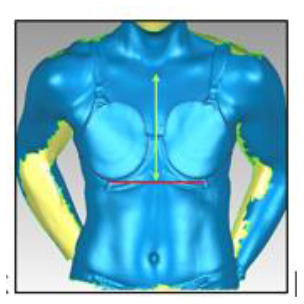

*Unless otherwise stated, all measurements were taken on each participant's right and left breast and the mean value was recorded*.

Eleven breast characteristics (including eight breast size and three breast position measurements; described in Table 3) were calculated from the scanned images using Geomagic Studio^®^ software (Version 12; 3DSystems, South Carolina, USA). These measurements were selected because they were deemed relevant to the design of military body armour based on recommendations for other torso-borne female equipment items, including the design of sports bras (McGhee and Steele, 2011; Zhou et al., 2013; Coltman et al., 2015, 2017a) and breast protective equipment (Brisbine et al., 2020a). Linear anthropometric measurements (such as the Sternal Notch to Nipple Distance) and three-dimensional measurements (such as Breast Volume and Surface Area) have previously been found to be accurate and valid when derived from three-dimensional scans (Paquette et al., 2000; Losken et al., 2005; Han et al., 2010; Qi et al., 2011; Yip et al., 2012). All calculations were completed by one researcher [BRB], who had high reliability in deriving these measurements (all ICC > 0.982; *p* < 0.001). Details of how each measurement was calculated are provided in Table 3.

### Perceived Body Armour Fit

Participants were asked to rank the overall fit of their current-issue body armour system (TBAS V4.4 Tier 2 or Tier 3) on a 5-point scale: 1 = *way too small/short/tight*; 2 = *too small/short/tight*; 3 = *good fit*; 4 = *too large/long/loose*; and 5 = *way too large/long/loose*. The responses *way too small/short/tight* and *too small/short/tight* were combined to form the category “too small” and responses *too large/long/loose* and *way too large/long/loose* were combined to form the category “too large” to create a categorical dependent variable with three groups. This question was from a larger 59-item questionnaire designed to explore the fit and function of current issue ADF body armour, which is described in more detail by Coltman et al. (2020). In brief, the 59-item questionnaire was developed following a focus group conducted with female soldiers (*n* = 8) and in consultation with subject matter experts within the Australian Army. The face validity and readability of the questionnaire were tested with personnel within the Defence Science and Technology Group and Australian Army. The questionnaire was published on a University of Canberra Qualtrics account (v0217; Provo, UT).

### Statistical Analyses

Descriptive statistics were calculated for all torso and breast characteristics listed in Tables 2, 3, including mean, standard deviation, 95% confidence interval and range. The frequency of participant responses to the perceived overall body armour fit (i.e., too small, good fit, and too large) was also calculated. A series of one-way ANOVAs were then performed to compare the mean value of all normally distributed anthropometric measurements (*n* = 12) characterising the torso and breast among those participants who reported their body armour was “too small,” a “good fit,” or “too large.” Games-Howell *post-hoc* analyses were then conducted to determine where any difference lay. For those measurements that did not meet the assumption of normality (*n* = 10), differences in mean rank were similarly assessed using a Kruskal-Wallis H-Test. Dwass-Steel-Critchlow-Fligner *post-hoc* analyses were then conducted to determine where any differences lay. Although multiple statistical tests were conducted, increasing the chance of incurring an error, no adjustment to the alpha level was deemed necessary given the exploratory nature of the study and the low cost associated with incurring a Type I error (Sinclair et al., 2013). Statistical analyses were performed in SPSS (Version 23, IBM Statistics, Chicago, USA) with an alpha level of *p* < 0.05.

## Results

### Torso Characteristics and Perceived Fit

Normative data on the torso characteristics of the participants are presented in Table 4. Comparisons of the mean value or mean rank of the torso characteristics among participants who reported that their body armour was “too small” (*n* = 14; 14%), a “good fit” (*n* = 30; 31%) or “too large” (*n* = 53; 55%) are presented in Table 5. There was a significant difference in both Stature [*f*_(2)_ = 3.715, *p* = 0.035] and Suprasternale Height [*f*_(2)_ = 3.899, *p* = 0.03] between participants who rated the fit of their body armour as good compared to those who rated it as too large, whereby female soldiers who rated the fit as too large were, on average, shorter than female soldiers who rated the fit as good. Chest Breadth [*f*_(2)_ = 11.547, *p* < 0.001] and Waist Circumference [$\chi_{\left(2\right)}^{2}$ = 15.77, *p* < 0.001] also differed significantly among those who rated the fit of their body armour as too small compared to too large, and too small compared to a good fit. Female soldiers who rated the fit as too small had, on average, larger torso breadths and circumferences than female soldiers who rated the fit as either good or too large. Similarly, Mass [$\chi_{\left(2\right)}^{2}$ = 12.85, *p* = 0.002], Chest Depth [*f*_(2)_ = 4.872, *p* = 0.014], Neck Circumference [$\chi_{\left(2\right)}^{2}$ = 10.20, *p* = 0.006] and chest circumference measures of OBCC [$\chi_{\left(2\right)}^{2}$ = 11.660, *p* = 0.003] and UBCC [$\chi_{\left(2\right)}^{2}$ = 9.16, *p* = 0.01] were also significantly larger, on average, in female soldiers who rated the fit as too small compared to those who rated the fit as too large. There was no significant difference in participants' ratings of fit for Waist Height [$\chi_{\left(2\right)}^{2}$ = 4.33, *p* = 0.115], Hip Circumference [$\chi_{\left(2\right)}^{2}$ = 5.78, *p* = 0.056], Front Length [*f*_(2)_ = 2.290, *p* = 0.112], Biacromial Breadth [*f*_(2)_ = 0.125, *p* = 0.883] or Iliocristale Height [*f*_(2)_ = 1.760, *p* = 0.188].

**Table 4:** Torso and breast characteristic data (mean ± standard deviation, confidence interval, and range) of the study participants (*n* = 97) are shown.

**Anthropometric characteristic**	**Mean ± SD**	**Mean ± SD**	**95% Confidence interval (lower bound, upper bound)**	**Range**
	**AWAS study**	**Current study**		
Stature (cm)	165.4 ± 6.1	165.3 ± 6.3	164, 166.5	152.6–180.5
Body Mass (kg)	66.0 ± 9.7	69.6 ± 10.5	67.5, 71.6	49.7–98.7
Iliocristale Height (cm)	100.9 ± 4.8	100.9 ± 4.8	99.9, 101.8	90.8–116.5
Waist Height (cm)	105.6 ± 4.8^a^	106.0 ± 5.5	104.9, 107.1	96.1–138.4
Suprasternale Height (cm)	134.1± 5.4	134.5 ± 5.4	133.4, 135.5	123.2–148.7
Front Length (cm)	33.2 ± 2.2	33.7 ± 2.3	33.2, 34.1	27.8–41.1
Chest Depth (cm)	—^b^	38.4 ± 1.7	27.4, 28.1	34.9–44.8
Chest Breadth (cm)	27.4 ± 2.3	27.8 ± 1.8	38.0, 38.7	23.4–32.6
Bi-acromial Breadth (cm)	37.3 ± 1.7	37.6 ± 1.6	37.3, 38.0	34.3–42.6
Neck Circumference (cm)	32.9 ± 2.0	32.8 ± 1.8	32.4, 33.1	29.6–37.5
Waist Circumference (cm)	83.7 ± 9.0^c^	74.7 ± 6.8	73.3, 76.0	62.1–98.5
Hip Circumference (cm)	99.3 ± 6.5	102.8 ± 6.6	101.5, 104.1	91.3–122.5
Over-Bust Chest Circumference (cm)	90.7 ± 7.5	89.7 ± 8.6	88.0, 91.4	34.1–109.0
Under-Bust Chest Circumference (cm)	76.5 ± 5.9	76.9 ± 5.6	75.8, 78.1	67.4–95.2
Breast Volume (mL)	—	365.2 ± 167.7	331.8, 398.6	103.6–885.8
Breast Surface Area (cm^2^)	—	443.9 ± 104.1	423.2, 464.6	250.5–706.6
Anterior Breast Projection (mm)	—	43.5 ± 10.3	41.5, 45.6	23.2–71.2
Breast Length (mm)	—	151.7 ± 14.8	148.8, 154.7	115.2–183.6
Breast Width (mm)	—	157.9 ± 17.3	154.5, 161.3	123.7–204.8
Sternal Notch to Nipple Distance (mm)	—	183.8 ± 19.5	179.9, 187.7	95.8–228.3
Sternal Notch to Superior Breast Distance (mm)	—	57.7 ± 10.8	55.6, 59.8	31.8–82.7
Sternal Notch to Inferior Breast Distance (mm)	—	209.4 ± 18.4	205.8, 213.1	164.3–266.3

*Normative data of female soldiers from the Australian Warfighter Anthropometric Survey (AWAS) are shown in the first column for comparison purposes (Edwards et al., 2014)*.

a *AWAS measurement of waist height was from the 10th rib, whereas the current study calculated Waist Height at the narrowest point between the 10th rib and the iliac crest*.

b *Although AWAS measurement of chest depth was collected, the measurement protocol was substantially different and comparative data have therefore been omitted*.

c *Waist Height was taken at different levels between studies as described in^a^, affecting the Waist Circumference measures*.

*—indicates measurements that were not collected in AWAS*.

**Table 5:** Torso characteristic data compared between the three fit groups (too small, good fit, and too large) using One-Way ANOVA (normally distributed data; difference in group means) or Kruskal–Wallis (non-normally distributed data; difference in mean rank).

**Torso characteristic**	**Fit**	***N***	**Mean**	**SD**	**SIG**	**vs. good fit**	**vs. too large**
Stature (cm)	Too small	14	167.2	7.9595	0.035^*^	0.997	0.318
	Good fit	30	167.0	4.8955			0.028^*^
	Too large	53	163.8	6.23			
Body Mass (kg)	Too small	14	78.0	10.241	0.002^*^	0.092	0.001^*^
	Good fit	30	71.0	10.4861			0.214
	Too large	53	66.5	9.1836			
Iliocristale Height (cm)	Too small	14	102.1	5.93	0.188	—	—
	Good fit	30	101.7	3.61			—
	Too large	52	100.0	4.99			
Waist Height (cm)	Too small	14	107.3	5.5	0.115	—	—
	Good fit	30	106.6	3.52			—
	Too large	53	105.4	6.42			
Suprasternale Height (cm)	Too small	14	136.1	6.57	0.03^*^	1	0.307
	Good fit	30	136.0	4.07			0.023^*^
	Too large	53	133.2	5.46			
Front Length (cm)	Too small	14	33.9	1.15	0.112	—	—
	Good fit	30	34.3	2.17			—
	Too large	52	33.2	2.58			
Chest Depth (cm)	Too small	14	39.5	1.99	0.014^*^	0.418	0.037^*^
	Good fit	30	38.7	1.55			0.081
	Too large	53	37.9	1.58			
Chest Breadth (cm)	Too small	14	29.6	1.58	<0.001^*^	0.004^*^	<0.001^*^
	Good fit	30	27.7	1.84			0.557
	Too large	53	27.3	1.56			
Bi-acromial Breadth (cm)	Too small	14	37.8	1.64	0.883	—	—
	Good fit	30	37.7	1.73			—
	Too large	53	37.6	1.6			
Neck Circumference (cm)	Too small	14	33.9	1.54	0.006^*^	0.124	0.005^*^
	Good fit	30	32.9	1.84			0.349
	Too large	53	32.4	1.73			
Waist Circumference (cm)	Too small	14	80.9	5.6	<0.001^*^	0.026^*^	<0.001^*^
	Good fit	30	75.3	7.49			0.396
	Too large	53	72.7	5.66			
Hip Circumference (cm)	Too small	14	107	7.71	0.056	—	—
	Good fit	30	103	7.12			—
	Too large	53	101.5	5.45			
Over-Bust Chest Circumference (cm)	Too small	14	96.0	6.6443	0.003^*^	0.068	0.002^*^
	Good fit	30	90.5	7.1124			0.485
	Too large	53	87.6	9.1305			
Under-Bust Chest Circumference (cm)	Too small	14	80.3	4.4869	0.01^*^	0.111	0.006^*^
	Good fit	30	77.7	6.7732			0.592
	Too large	53	75.7	4.7941			

*The third last column provides the p-value for the main effects of fit group on each torso characteristic. The final 2 columns provide p-values of the pairwise comparisons between the three fit groups, as determined through post-hoc analysis*.

* *represents significance at p < 0.05. For variables that were found to have no significant difference between fit groups, post-hoc tests were not conducted and the corresponding cells were marked with a long dash*.

### Breast Characteristics and Perceived Fit

Normative data on the breast characteristics of the participants are presented in Table 4. Comparisons of the mean value or mean rank of the breast characteristics among participants who reported their body armour was “too small” (*n* = 14), a “good fit” (*n* = 30) or “too large” (*n* = 53) are presented in Table 6. Breast Surface Area [*f*_(2)_ = 4.596, *p* = 0.018], Breast Width [*f*_(2)_ = 6.150, *p* = 0.005], and Sternal Notch to Nipple Distance [$\chi_{\left(2\right)}^{2}$ = 12.32, *p* = 0.002] differed significantly among those who rated the fit of their body armour as too small compared to too large and too small compared to a good fit. Female soldiers who rated the fit as too small had, on average, larger breast sizes, shapes and positions than female soldiers who rated the fit as either good or too large. Similarly, Breast Volume [$\chi_{\left(2\right)}^{2}$ = 6.43, *p* = 0.04], Anterior Breast Projection [$\chi_{\left(2\right)}^{2}$ = 6.33, *p* = 0.042], Breast Length [*f*_(2)_ = 4.923, *p* = 0.013] and Sternal Notch to Inferior Breast Distance [*f*_(2)_ = 4.047, *p* = 0.027] were significantly larger, on average, in female soldiers who rated the fit of their body armour as too small compared to those who rated the fit as too large. There was no significant difference in participants' rating of fit for Sternal Notch to Superior Breast Distance [*f*_(2)_ = 0.859, *p* = 0.433].

**Table 6:** Breast characteristic data compared between the three fit groups (too small, good fit, and too large) using One-Way ANOVA (normally distributed data; difference in group means) or Kruskal–Wallis (non-normally distributed data; difference in mean rank).

**Breast characteristic**	**Fit**	***N***	**Mean**	**SD**	**SIG**	**vs. good fit**	**vs. too large**
Breast Volume (mL)	Too small	14	503.0	225.3185	0.04^*^	0.064	0.039^*^
	Good fit	30	344.5	152.4872			1
	Too large	53	340.5113	142.4827			
Breast Surface Area (cm^2^)	Too small	14	528.8143	116.4542	0.018^*^	0.044^*^	0.018^*^
	Good fit	30	435.46	102.9021			0.913
	Too large	53	426.2811	91.6439			
Anterior Breast Projection (mm)	Too small	14	50.85	11.8747	0.042^*^	0.062	0.044^*^
	Good fit	30	42.2467	9.9361			0.977
	Too large	53	42.3509	9.3534			
Breast Length (mm)	Too small	14	162.3857	13.9594	0.013^*^	0.064	0.013^*^
	Good fit	30	151.4233	15.1327			0.773
	Too large	53	149.1226	13.8933			
Breast Width (mm)	Too small	14	173.3	18.1845	0.005^*^	0.02^*^	0.006^*^
	Good fit	30	156.4133	17.3884			0.89
	Too large	53	154.6679	14.9703			
Sternal Notch to Nipple Distance (mm)	Too small	10	203.01	14.9471	0.002^*^	0.006^*^	0.002^*^
	Good fit	25	181.724	16.6891			0.989
	Too large	49	180.9408	19.7643			
Sternal Notch to Superior Breast Distance (mm)	Too small	14	61.2214	12.9773	0.433	—	—
	Good fit	30	58.1867	9.0009			—
	Too large	53	56.4868	11.0649			
Sternal Notch to Inferior Breast Distance (mm)	Too small	14	223.6214	22.1102	0.027^*^	0.113	0.029^*^
	Good fit	30	209.61	16.6556			0.549
	Too large	53	205.6038	16.7754			

*The third last column provides the p-value for the main effects of fit group on each breast characteristic. The final 2 columns provide p-values of the pairwise comparisons between the three fit groups, as determined through post-hoc analysis*.

* *represents significance at p < 0.05. For variables that were found to have no significant difference between fit groups, post-hoc tests were not conducted and the corresponding cells were marked with a long dash*.

## Discussion

This is the first published study to provide normative data on the torso and breast size and shape of female soldiers. These soldiers displayed a wide range of anthropometric characteristics that must be considered when sizing and designing body armour. Sixteen of the twenty-two measurements assessed were significantly associated with the soldiers' ratings of body armour fit, suggesting a link between breast and torso characteristics and the overall perceived fit of body armour systems by female soldiers. The implications of these findings in terms of the design and sizing of current-issue body armour systems are discussed below.

Given that body armour systems are torso borne, the size and shape of the torso is a key consideration when designing body armour systems to closely interface with the human body. Among study participants, torso characteristic data were similar to all comparable measurements previously reported by the AWAS for ADF female soldiers (Edwards et al., 2014). Large variations, however, were observed in individual measurements, evident in the large standard deviations (Table 4). This large variability highlights the range of shapes and sizes that body armour systems should be designed to accommodate, as well as sex-related differences in torso dimensions compared to male AWAS data (Edwards et al., 2014). For example, female soldiers in the present study had substantially narrower chests (27.8 vs. 30.5 cm AWAS; Chest Breadth) and smaller waists (74.7 vs. 92.4 cm AWAS; Waist Circumference) but larger hips (102.8 vs. 99.7 cm AWAS; Hip Circumference) when compared to male AWAS data (Edwards et al., 2014). Given the limited, unisex sizing range of current issue armour, it is unsurprising that many females have reported to be wearing ill-fitting body armour (Coltman et al., 2020).

Consistent with our hypothesis, several torso measurements were significantly associated with a poor fit. Participants who perceived their body armour to be too large (*n* = 53) were more likely to be shorter in Stature and Suprasternale Height (Table 5) compared to those who perceived their body armour to be a good fit (*n* = 30), suggesting the length of the in-service body armour system may be too long for many users. Body armour is designed to be positioned superiorly at the level of the suprasternal notch. As armour length increases relative to overall height and height of the suprasternal notch, it is increasingly likely to interfere with trunk mobility and task performance and, therefore, perceptions of armour fit (Molloy et al., 2020; Coltman et al., 2021a, 2022). Conversely, participants who reported their body armour to be too small (*n* = 14) had a significantly greater Mass, chest circumference (OBCC and UBCC), Chest Depth, Chest Breadth, Waist Circumference, and Neck Circumference compared to those who perceived their body armour to be too large (*n* = 54). This either suggests that the body armour system is not wide enough to accommodate large torso circumferences or that the mechanism to tighten the system does not have sufficient adjustment capacity. Designers of body armour for women should pay particular attention to ensure adjustment points on the front and rear carriers, including the shoulder straps and cummerbund, can cater for the variability in chest curvature of women's torsos due to their additional breast tissue. Ultimately, military organisations should consider developing body armour systems that are better suited to the range of female soldier torso sizes, both large and small (Wen and Shih, 2020). Such systems are likely to improve perceptions of fit and have important implications for task performance and efficiency in the field (Mitchell et al., 2010; Choi et al., 2019; Coltman et al., 2021a). Issuance procedures (e.g., sizing and allocation of systems) and education (e.g., training on system use) should also be updated to accompany the introduction of any new equipment because previous research has identified that these factors contribute to reports of dissatisfaction among female body armour users (Coltman et al., 2021a).

Clearly, the breasts of female soldiers are substantially different to male soldiers, and this disparity can affect body armour fit. The mean Breast Volume reported in this study for female soldiers (365 ml) was consistent with mean Breast Volume for women from an athletic population (401.7 ml; Brisbine et al., 2020a), but smaller than that previously reported for the general female population (653 ml left and 647 ml right breast; Coltman et al., 2017a). This smaller average breast size of the female soldiers is likely to be a function of the younger age (mean: 25.6 years) and lower body mass index (mean: 25.4 kg/m^2^) of the study participants compared to the general population (Edwards et al., 2014; Coltman et al., 2017a). There was, however, a wide range of breast sizes (see Table 3). This diversity in breast size has important implications for armour design. For example, female soldiers with large breasts are likely to benefit from body armour systems designed with space to accommodate additional breast tissue, although such space would not be needed for soldiers with small breasts. Given the similarity of average breast size between participants who rated their armour as a good fit (344.5 mL; *n* = 14; 14%) and too large (340.5 mL; *n* = 30; 31%) compared to those who rated their armour as too small (503.0 mL; *n* = 53; 55%; Table 6), it would appear that female soldiers with a breast size of medium or large (formally classed as 350 mL and above; Coltman et al., 2017b) are most affected by sizing of body armour systems around the chest. This represents a substantial portion of the female soldier population, thus highlighting the importance of using anthropometric data to inform future armour design.

In addition to breast size, breast shape characteristics, such as Breast Surface Area and Anterior Breast Projection, will influence the overall fit of a torso borne body armour system. Given the range of breast projection distances documented in the current study (23–71 mm), women with breasts that protrude further anteriorly from the chest wall are likely to experience greater difficulty achieving conformity between a body armour system and their torso (Coltman et al., 2021b). The resultant compression and deformation of the breast when wearing body armour, which is exaggerated in women with large breasts (McGhee and Steele, 2020), is likely to have negative functional and protection impacts (e.g., difficulty breathing, limited ROM, exposed lateral breast tissue, and compromised positioning of vital torso protection). Consequently, our findings corroborate previous reports that occupational body armour does not adequately accommodate the full range of female breast characteristics (Coltman et al., 2020, 2021a; Malbon et al., 2020; Niemczyk et al., 2020). It was therefore unsurprising that Breast Volume, Breast Surface Area and Anterior Breast Projection were each associated with the perception of poor body armour fit (Table 6). All three breast measures were significantly larger in female soldiers who perceived their body armour to be too small compared to those who perceived it to be too large, with Breast Surface Area additionally differing between those who perceived their body armour to be too small and a good fit. As previous research has demonstrated the capacity of body armour to cause mild restrictive ventilatory impairment in male soldiers (Armstrong and Gay, 2016; Armstrong et al., 2019), reports of difficulty breathing due to excessive compression of the breasts and chest in the present study are likely magnified as breast size increases. Further research, however, is required to confirm this notion. Similarly, experiences of body armour being too loose around the waist to accommodate additional tissue in the upper chest may be further associated with breast size and shape variations amongst female soldiers (Coltman et al., 2021a). In both instances, design modifications to body armour have the potential to reduce breathing restrictions and, in turn, minimise discomfort and impairments to performance (Armstrong and Gay, 2016). Body armour system weight and load carriage have previously been associated with expiratory flow limitations (Armstrong et al., 2019). Furthermore, excessive weight of body armour has been reported as the third-most disliked characteristic of current-issue body armour by female soldiers (Coltman et al., 2022). Therefore, a lighter body armour system, one with smaller sizing dimensions, is likely to afford similar benefits to respiratory function.

Female soldiers in the present study also displayed a wide variety of breast positions relative to their torso. This was illustrated by a wide range of Sternal Notch to Nipple Distances (95.8–228.3 mm) and breast position measures, whereby the superior border of the breast was found to sit just 3 cm below the sternal notch for some soldiers and more than 5 cm lower for others. Body armour should be positioned at the level of the sternal notch to ensure sufficient coverage of the vital thoracoabdominal organs (Laing and Jaffrey, 2019). Knowledge of breast position is therefore crucial when designing body armour for female soldiers. Importantly, female soldiers whose breasts were situated lower on their torso were more likely to perceive their body armour system fit as too small. Coupled with other breast size and shape measures, breast position data indicate where potential plate shape changes or additional adjustability features need to be incorporated into a body armour system to better accommodate female breasts. Police body armour manufacturers have explored thermal forming and darting to provide a female-specific shape to soft ballistic plates (Basich, 2007). There is similar support in the literature for a contoured plate based on three-dimensional torso scans or anthropometry of female wearers (Boussu and Bruniaux, 2012; Cichocka et al., 2014; Mahbub et al., 2014; Abtew et al., 2018). The concept of a formed plate also warrants consideration for military body armour applications. The benefits of contouring rigid military hard ballistic plates, however, are not fully understood, and it is unknown how mobility and ballistic performance may be influenced by any changes. Female soldiers have previously reported that body armour is either too tight over the breasts to fit correctly around the waist or too loose around the waist to fit correctly over the breasts (Coltman et al., 2021a). An average difference of 15 cm was reported between mean chest circumference (OBCC; 89.7 cm) and mean Waist Circumference (74.7 cm) in this study, suggesting that incorporating multiple adjustments points on body armour systems is warranted. Any form changes, however, must be evaluated against protection implications because protection remains the primary function of military body armour systems (Laing and Jaffrey, 2019).

Fit and function issues associated with body armour are further exacerbated by wearing a bra because a bra can change breast shape and limit breast deformability under a hard plate. Moreover, bras function to lift and support the breasts to reduce pain and discomfort associated with breast movement (Scurr et al., 2014; Brisbine et al., 2020b), whereas body armour places downwards pressure on the breasts, effectively acting against the support provided by a bra (Niemczyk et al., 2020). Therein lies an additional challenge in designing body armour suitable for female soldiers with large breasts. That is, large breasts require more upwards support from a bra (thus limiting deformability) yet are more susceptible to the downward compression from body armour that may act to counteract this support. Bras can also be a major source of discomfort for female wearers of body armour because of the way body armour affects the fit and form of otherwise well-fitting, supportive bras (Niemczyk et al., 2017, 2020; Burbage et al., 2021). Furthermore, body armour can limit the type of bra a female soldier can wear. Compression-style sports bras, which are commonly wire-free and function to reduce anterior protrusion of the breast, are the most commonly worn type of bra among Australian (Coltman et al., 2021b) and British female soldiers (Burbage et al., 2021). Compression-style bras, however, do not provide sufficient breast support for women with medium-large breast sizes (McGhee et al., 2008), who comprised 44% (*n* = 43) of the study population. Researchers examining police body armour have suggested that designing a supportive bra to specifically integrate with body armour may be an effective short-term solution (Malbon et al., 2020; Niemczyk et al., 2020). Such a bra should be a wire-free design that reduces anterior breast protrusion while distributing breast tissue to improve the fit of body armour over the breasts and alleviate discomfort associated with current bra-armour incompatibility (Malbon et al., 2020; Coltman et al., 2021b).

The findings of the current study must be considered in light of their limitations. Although this study was the first to provide normative data on female soldiers' torso and breast characteristics, no objective measures of body armour fit or performance were collected. Therefore, breast characteristics could only be associated with the fit of body armour reported by the female soldiers. Participant perceptions have been used in other research evaluating protective equipment (Park and Hahn, 2014) and therefore are deemed to be a valuable metric for assessing overall fit in the present study. Future research, however, is recommended to incorporate both subjective and objective measures related to body armour fit, including static, dynamic, occupation-specific and cognitive fit (Stirling et al., 2020). Additionally, the sizes of the body armour systems issued to the participants were not recorded; TBAS Tier 2 is only available in one size, but TBAS Tier 3 is available in four sizes (S-XL), which may account for some differences in participant ratings of subjective fit. Participants were not asked to bring their body armour system to the test session and most participants were unaware of the size of the body armour that they had been issued, preventing collection of these data. As this study focused exclusively on the anthropometry, design, and sizing requirements of female soldiers, it is also recommended that future research similarly profile the male torso to assess potential fit and form issues experienced by male soldiers and to better target design and sizing improvements to the entire soldier population. Fundamentally, any design changes must be considered against known protection requirements, including vital thoracoabdominal organ protection.

## Conclusion

Normative data characterising the torso and breasts of 97 female soldiers highlight the variation in anthropometric dimensions that body armour systems must cater for, as well as the implications of the varied torso and breast sizes and shapes for perceived body armour fit. Future body armour systems should cater for female soldiers' physical diversity by developing an expanded sizing range and female-specific design features to improve perceptions of fit. Improved perceptions of fit will, in turn, reduce movement interference, discomfort, and barriers to performance in the field. Any modifications to body armour should be informed by anthropometric data representing female soldiers and aim to ensure that vital thoracoabdominal organ protection recommendations are maintained.

## Data Availability Statement

The datasets presented in this article are not readily available because participants of this study did not agree for their data to be shared publicly. Requests to access the datasets should be directed to Celeste.Coltman@Canberra.edu.au.

## Ethics Statement

The studies involving human participants were reviewed and approved by Defence Science and Technology Group Low Risk Ethics Panel, Department of Defence, Australia. The participants provided their written informed consent to participate in this study.

## Author Contributions

CC was the primary investigator. She was responsible for the study design, recruiting the participants collecting, analysing and interpreting the data, co-writing the first full draft of the manuscript, and approving the final version of the manuscript. BB was responsible for analysing and interpreting the data, co-writing the first full draft of the manuscript with the primary investigator, and approving the final version of the manuscript. RM was responsible for helping to develop the study design, recruiting the participants, assisting to interpret the data, providing feedback on versions of the manuscript, and approving the final version of the manuscript. JS was responsible for helping to develop the study design, assisting to interpret the data, providing substantial feedback on versions of the manuscript and approving the final version of the manuscript. All authors contributed to the article and approved the submitted version.

## Funding

This study was funded by the Defence Science and Technology Group, Department of Defence, Australia under a Defence Science Partnering Deed (reference number 7974) as part of an ongoing effort to improve the fit, form, and function of equipment, to accommodate the diverse body shapes of Australian soldiers.

## Conflict of Interest

RM was an employee of Defence Science and Technology Group, Department of Defence, Australia. The remaining authors declare that the research was conducted in the absence of any commercial or financial relationships that could be construed as a potential conflict of interest.

## Publisher's Note

All claims expressed in this article are solely those of the authors and do not necessarily represent those of their affiliated organizations, or those of the publisher, the editors and the reviewers. Any product that may be evaluated in this article, or claim that may be made by its manufacturer, is not guaranteed or endorsed by the publisher.
